# The archaeological ensemble from Campoverde (Agro Pontino, central Italy): new constraints on the Last Interglacial sea level markers

**DOI:** 10.1038/s41598-018-36111-x

**Published:** 2018-12-13

**Authors:** F. Marra, C. Petronio, P. Ceruleo, G. Di Stefano, F. Florindo, M. Gatta, M. La Rosa, M. F. Rolfo, L. Salari

**Affiliations:** 1 Istituto Nazionale di Geofisica e Vulcanologia, Via di Vigna Murata 605, 00143 Rome, Italy; 2 Università di Roma “Sapienza”, Dipartimento di Scienze della Terra, P.le Aldo Moro 5, 00185 Rome, Italy; 3 Freelance, Tivoli, (Rome) Italy; 4 Università di Roma “Tor Vergata”, Department of History, Humanities and Society, Via Columbia 1, 00133 Rome, Italy; 5 University of York, Department of Archaeology, The King’s Manor, York, UK; 6 Ecomuseo dell’Agro Pontino, Via G.B. Vico 15, Latina, Italy

## Abstract

We present a combined geomorphological and biochronological study aimed at providing age constraints to the deposits forming a wide paleo-surface in the coastal area of the Tyrrhenian Sea, south of Anzio promontory (central Italy). We review the faunal assemblage recovered in Campoverde, evidencing the occurrence of the modern fallow deer subspecies *Dama dama dama*, which in peninsular Italy is not present before MIS 5e, providing a post-quem terminus of 125 ka for the deposit hosting the fossil remains. The geomorphological reconstruction shows that Campoverde is located within the highest of three paleosurfaces progressively declining towards the present coast, at average elevations of 36, 26 and 15 m a.s.l. The two lowest paleosurfaces match the elevation of the previously recognized marine terraces in this area; we define a new, upper marine terrace corresponding to the 36 m paleosurface, which we name Campoverde complex. Based on the provided evidence of an age as young as MIS 5e for this terrace, we discuss the possibility that previous identification of a tectonically stable MIS 5e coastline ranging 10–8 m a.s.l. in this area should be revised, with significant implications on assessment of the amplitude of sea-level oscillations during the Last Interglacial in the Mediterranean Sea.

## Introduction

**Figure 1:**
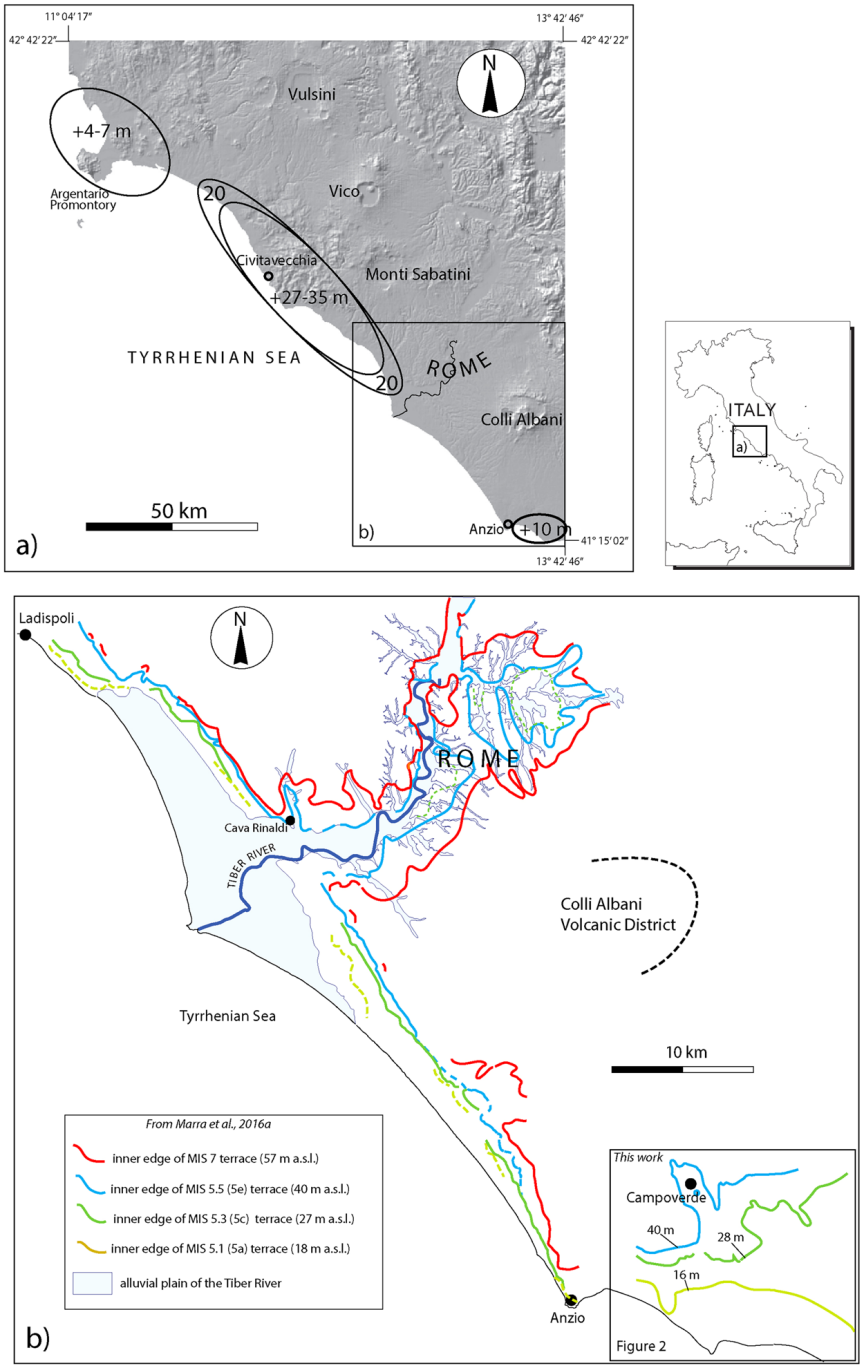
(**a**) Digital Elevation Map (TINITALY/01 square WA 6570, property of the Istituto Nazionale di Geofisica e Vulcanologia, Rome, http://tinitaly.pi.ingv.it/) of southern Tuscany and Latium regions showing the elevation (in m) of the coastal terrace of MIS 5e (also commonly reported as 5.5) in different sectors (from^15^. The direct, spatial relationship between the coastal uplifted sector, comprised between the Argentario and Anzio promontories, and the location of the volcanic districts of the Roman Province^7^ (and references therein) is highlighted. (**b**) Inner edges of the coastal terraces of MIS 7 through MIS 5a, reconstructed in the sector between Ladispoli and Anzio^5^. Inner edges and elevation (m a.s.l.) of a suite of three coastal terraces reconstructed in this work south of Anzio is also shown.

A large number of vertebrate bones and lithic artifacts was recovered from the subsurface sedimentary deposits affected by trench excavation, during work to reclaim and align the course of a small stream channel in Campoverde, ~10 km northeast of the town of Anzio in the Pontina plain (Agro Pontino) (Fig. 1). The archaeological findings were the subject of preliminary publications^1–4^, before being stored at the museum “Antiquarium Comunale” of Nettuno.

**Figure 2:**
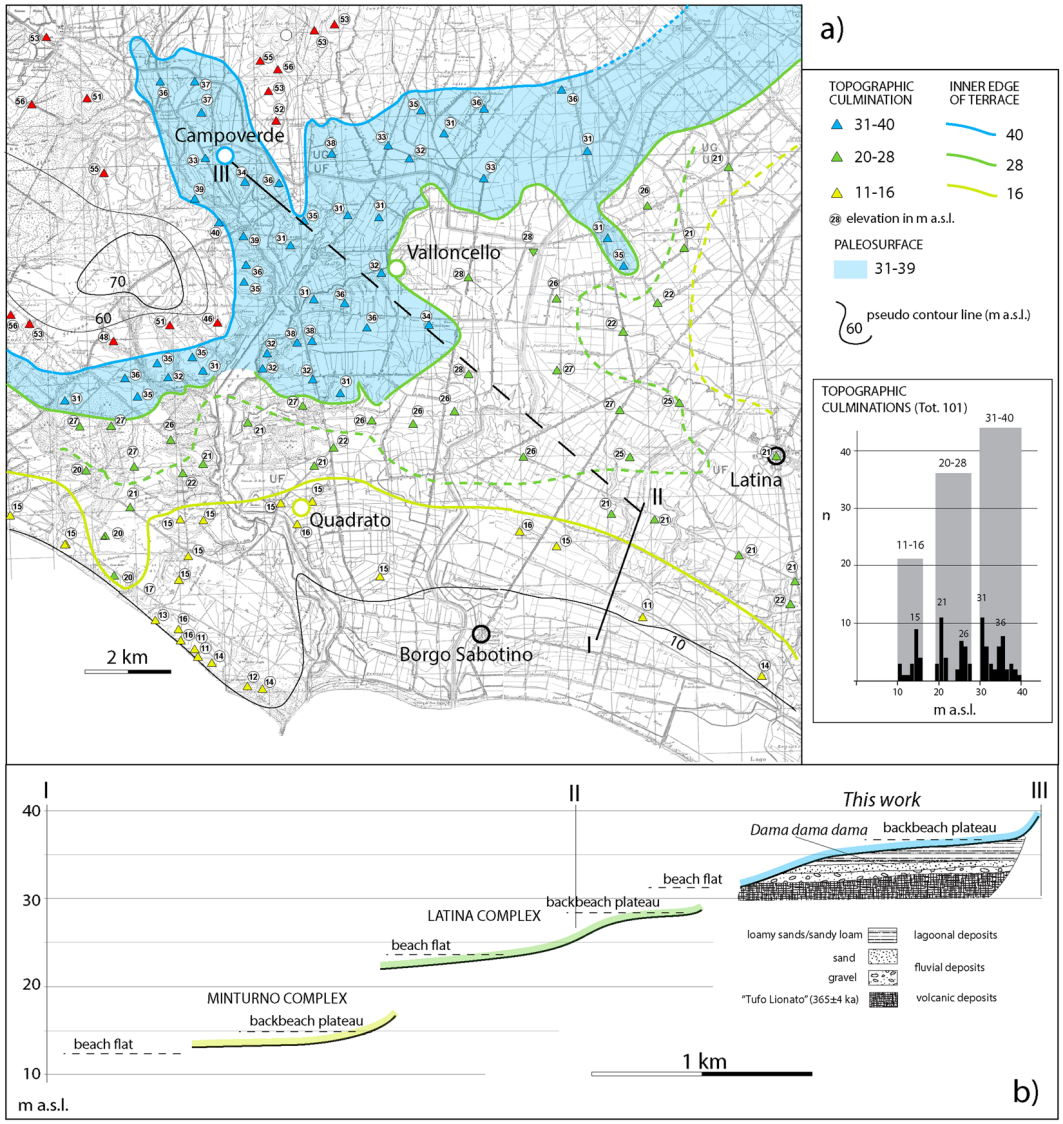
(**a**) Geomorphologic map and statistics. Three paleosurfaces ranging 11–16, 20–28, and 31–40 m a.s.l., are depicted. The topographic culminations identified in the IMG 1:25.000 maps are reported with triangles of different colors on the 1:10.000 topographic base by Regione Lazio (http://dati.lazio.it/catalog/it/dataset/carta-tecnica-regionale-1991), available under Creative Commons Attribution License (https://creativecommons.org/licens/by/4.0/). Each color is associated with an elevation range defining a paleosurface which, in turn, is established based on the statistical analysis. See text for further explanation. (**b**) Cross-section showing the three marine terraces recognized in this area: the lower Latina and Minturno complexes (description of the associated deposits by^17^ is provided in the text), and the higher Campoverde complex recognized in this study.

The area of Campoverde is a large level plateau, at elevations ranging 31–39 m a.s.l., partially dissected by fluvial incisions (Fig. 2). Recent work has evidenced the occurrence of a suite of paleosurfaces along the central coast of Latium, between Ladispoli and Anzio, identified as three coastal terraces at elevations of ca. 36 m, 26 m, and 15 m a.s.l. (Fig. 1b ^5^). These terraces are interpreted as reflecting combined isostatic (e.g.^6^) and volcano-tectonic uplift since 250 ka, interpunctuated by a minor phase of collapse around 100 ka^5^, linked with the presence of the volcanic districts of the Roman Magmatic Province^7^.

Indeed, the area where these terraces are recognized corresponds to the coastal reach facing the Monti Sabatini and Colli Albani volcanic districts (Fig. 1), which were active 800 ka through 86 ka^8,9^, and 600 ka though 36 ka^10^, respectively.

A ^40^Ar/^39^Ar age constraint of 129 ± 1 ka allowed correlation^11^ of the 36 m terrace with the highstand of (Marine Isotopic Stage) MIS 5e (125 ka^12^), in good agreement with previous geochronologic constraints achieved on the sedimentary deposits forming a terrace at higher elevation, ranging 51–56 m a.s.l., providing correlation with MIS 7^5,13^. Based on the strong age constraint for the 56 m and 36 m terraces, and lacking direct geochronologic constraints for the lower terraces at 26 m and at 15 m, they have tentatively been correlated^5^ with MIS 5c (100 ka) and MIS 5a (80 ka), respectively, following the principle of a staircase geometry (e.g.^14^).

In contrast with the widespread morphological evidence all along the coast to the north, the terrace at 36 m was not recognized southeast of Anzio, where related literature accounts for several sea-level indicators ranging from 10 to 6 m a.s.l., which are identified as the MIS 5e sea-level^15^ (and references therein). In the present work, we have performed a geomorphological study of the coastal area southeast of Anzio, and we have re-analyzed the faunal remains hosted at the Nettuno museum with the aim of providing biochronological constraints to the Campoverde paleosurface, in order to check its chronological relationships with the 36 m terrace north of Anzio, and with the sea-level indicators attributed to MIS 5e in this area.

Moreover, we have re-analyzed the lithic artifacts recovered along with the faunal remains in Campoverde, aiming to provide techno-typological and taphonomic information to support the paleontological and paleoenvironmental interpretations provided in this paper.

## Paleontological and Palethnological Investigations

The fossil remains and lithic artifacts originally recovered at Campoverde are housed in the Paleontological and Palethnological Collection of the “Antiquarium Comunale” in Nettuno (Rome). The bone remains and the lithic artifacts were collected in two distinct areas not far from each other (CV1 and CV2) from the sediment accumulated on the ground level during excavation of the irrigation channel. For this reason, apart for a few specimens collected *in situ*, it was not possible to establish the exact stratigraphic level for most finds^1^.

The mammal fossils from Campoverde have been reexamined and re-measured for the present work.

Abbreviations: L.: length; B. breadth; MTD: transverse diameter of diaphysis; MAPD: antero-posterior diameter of diaphysis; DTD: distal transverse diameter; DAPD: distal antero-posterior diameter; DB/L index: distal transverse diameter/length * 100; MB/L index: transverse diameter of diaphysis/length * 100.

The lithic collection from Campoverde is composed of 562 artifacts. The first sample of 245 artifacts was techno-typologically examined in the past^2–4^, while the remaining lithic industries have been analysed for this paper.

## Geomorphological Investigations

In the present study, paleosurfaces have been mapped following a well-established geomorphological approach^5,16^, based on the identification of a set of flat surfaces characterized by topographic culminations with elevation ranging through a few meters around a mean value. Selected topographic culminations of the reconstructed paleosurfaces were detected on the 1:25.000 topographic maps of Italy edited by Istituto Geografico Militare (IGM) (sheets: 158 I SO, 158 I SE, 158 II NO, 158 II NE, 158 II SE, 158 III NO, 158 IV SE). They include all the hilltops (i.e., each elevation point within a closed, 5 m spaced contour line) and other quasi-equivalent points within almost closed contour lines bordering plateau-like sectors. Distribution of the topographic culminations has been statistically analyzed in order to verify the occurrence of discrete elevation intervals corresponding to peaks of concentration, which can be assumed as the mean value for each paleosurface. The identification of the paleosurfaces implies a combined approach that integrates statistically significant concentrations of elevations around a mean value, and the morphologic evidence for the concentration of these elevation points within a finite area, as detected in the maps.

Due to the copyright covering the topographic maps by IGM, we have used the 1:10.000 technical maps by Regione Lazio (http://dati.lazio.it/catalog/it/dataset/carta-tecnica-regionale-1991), available under Creative Commons Attribution License (https://creativecommons.org/licenses/by/4.0/), as a topographic base in Fig. 2 of this paper.

## Geomorphological Reconstruction

Results of the geomorphological study are shown in Fig. 2a, where three very well-defined paleosurfaces at 11–16, 20–28, and 31–40 m a.s.l., are depicted in the coastal sector southeast of Anzio. The topographic culminations identified on the 1:25.000 maps are reported with triangles of different colors. Each color is associated with an elevation range defining a paleosurface which, in turn, is established based on the statistical analysis. Elevation ranges for the paleosurfaces are represented by grey boxes of cumulated frequency. Blue color shading is used in Fig. 2a for the 31–40 m paleosurface, while inner margins represented by solid colored lines are reported for the lower paleosurfaces, located closer to the coast.

The intermediate paleosurface is characterized by two separate peaks of concentration at 20–22 and 25–28 m a.s.l. (Fig. 2b). Similarly, the lowest paleosurface has a minor peak at 11 m and the main peak at 15 m a.s.l., and the highest one has two peaks at 31 and 36 m a.s.l. We interpret this feature as reflecting a coastal set represented by a beach flat (lowest elevation peak) and a backbeach plateau including the coastal plain (highest elevation peak), separated by the beach ridge, as illustrated in Fig. 2b.

Finally, the occurrence of a highest, less well preserved paleosurface is suggested by a series of topographic culminations ranging 51–58 m a.s.l. in the northwestern portion of this area. Given its limited extent at the margin of the investigated coastal sector, a possible inner margin for this paleosurface is not reported.

## The Latina and Minturno Complexes

Remarkably, the two lowest order paleosurfaces identified by the geomorphological study, at 20–28 m and 11–16 m a.s.l., match well with two marine terraces identified by previous physiographic studies^17^, termed the Latina complex and the Minturno complex, respectively (Fig. 2b).

The Latina complex consists of a dissected level plateau, 20 to 25 m a.s.l., mainly composed of well-sorted fine augite-rich sands with rare intercalations of clay, representative of a brackish/marine environment^17^. Borehole data in^17^ (Fig. 2b) indicated that at a depth of 12–15 m this homogeneous sand horizon is underlain by fossiliferous calcareous muds, of lagoonal origin, with peat intercalations. On the more level and less eroded, upper part of the Latina complex, the sands are covered by a layer of greenish clay to sandy clay loam, 1.5–3.0 m in thickness.

The Minturno complex as been defined^17^ as consisting of an elongated ridge 8 to 15 m a.s.l., and of a posterior dissected level area, 11 to 16 m a.s.l. (Fig. 2b). Textures of the deposits range from sandy clay loam to loamy coarse sand with sparse gravel, and they are interpreted as a beachridge system, overlying greenish lagoonal clay above which the beachridge migrated. These greenish, non-stratified clays also form the top of the deposits in the level area (Fig. 2b). Outcrop data show that the clays lie conformably on loams and subsequently gravels, forming a fining upwards sequence overall^17^.

Based on tentative correlation with known marine terrace sequences for Latium, a correspondence with the *Tyrrhenian II* stage (approximately 127 ka, corresponding to MIS 5e) was proposed for the Minturno complex in^17^, whereas only a relative, older age was assigned to the Latina complex. This correlation is derived from the elevations at which the raised beach deposits containing the warm exotic gastropod *Strombus bubonius* occur in northern Latium, namely 2–3 m (*STROMBUS III*, ~90 ka), 10–15 m (*STROMBUS II*, ~127 ka), and 18–25 m a.s.l. (*STROMBUS I*, ~200 ka)^18^. Subsequently, based on the definition of characteristic aminozones (i.e., based on amino acid geochronology) previous interpretations of three distinct *Strombus* levels occurring along the Latium coast have been rejected^19^, although correlation of the Minturno complex transgression with the MIS 5e interglacial of ca. 125 ka has been coinfirmed^19^. The MIS 5e terrace has been successively identified at varying elevations in the area comprised between Monte Argentario and Rome, ranging over 20–36 m a.s.l. due to differential tectonic uplift, and progressively descending to ~10 m in the stable sectors to the north and to the south^15,20^, see Fig. 1a). More recently^11^, have provided a ^40^Ar/^39^Ar age of 129 ± 1 ka on a primary volcanic layer intercalated in the deposits forming the 36 m terrace at Cava Rinaldi in the central coastal area (Fig. 1b), demonstrating its correlation with MIS 5e. Moreover, the 36 m terrace has been reconstructed^5^ over a wide coastal reach, showing that it homogeneously extends between Ladispoli and Anzio (Fig. 1b), revising previous inferences of varying elevation due to differential uplift rates along the coast. In contrast, it has also been shown^5^ that two lower, younger coastal terraces are present in this area, at an elevation of ca. 27 and ca. 16 m, which were previously interpreted as tectonically displaced portions of the MIS 5e terrace. No direct geochronologic constraint has been so far provided on these lower terraces, which have been tentatively correlated with MIS 5c and MIS 5a, respectively (Fig. 1b ^5^).

## Campoverde Complex

**Figure 3:**
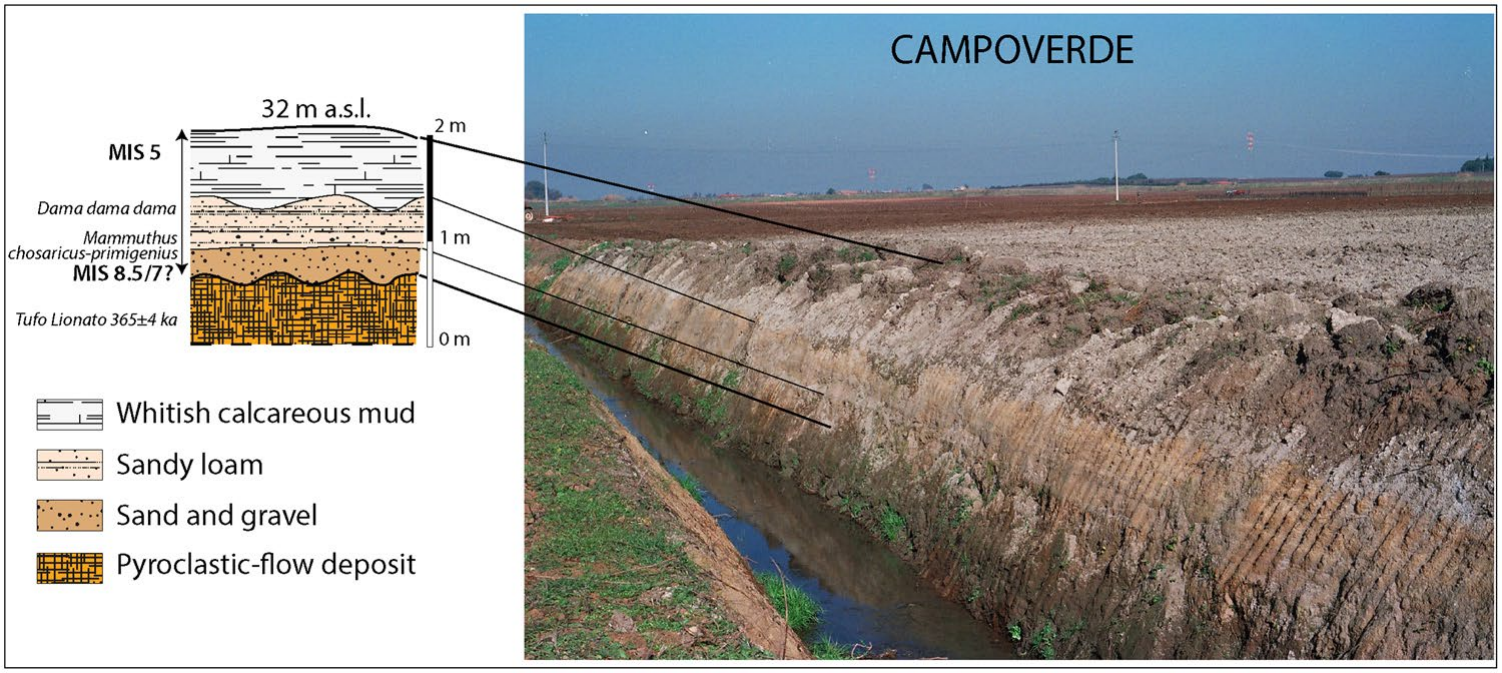
Outcrop of the deposits forming the Campoverde complex (photo by authors). See text for comments.

Excavations to rectify the stream channel exposed a ca. 2.5 m high section at Campoverde, in the middle of the flat area at around 36 m a.s.l. (Figs 2 and 3). The trench cut exposed a fining-upwards sedimentary succession unconformably overlying the pyroclastic-flow deposit of Tufo Lionato (Lower Flow Unit of the Villa Senni Eruption Cycle, 365 ± 4 ka^21^). A gravelly sand sediment of fluvial origin rich in reworked volcanics represents the basal coarse deposit of the sedimentary sequence, which grades upward into a loamy sand deposit, followed by whitish calcareous muds of a brackish/lagoonal environment. These features allow the sedimentary sequence at Campoverde to be interpreted as an aggradational succession deposited in response to sea-level rise during a glacial termination, according to the definition by^13^. It is indeed a transgressive deposit which, by analogy with the Latina and Minturno complexes defined in this area^17^, we name the Campoverde complex (Fig. 2b).

## Revision of the Faunal Assemblage

The following taxa have been identified in the faunal assemblage of Campoverde (Table 1).

**Table 1:** Summary of paleontological revisions to the Campoverde mammal assemblage.

Mazza *et al*. 1992	this work
*Elephas (Palaeoloxodon) antiquus*	*Palaeoloxodon antiquus*
*Mammuthus primigenius* (archaic form)	*Mammuthus* ex gr. *chosarichus-primigenius*
*Ursus* sp. (cf. *U. deningeri-spelaeus* group)	*Ursus spelaeus*
*Canis* sp. (cf. *C. mosbachensis*)	*Canis lupus*
*Equus caballus*	*Equus ferus*
*Equus hydruntinus*	*Equus hydruntinus*
*Stephanorhinus* sp. (cf. *S. hundsheimensis*)	*Stephanorhinus* sp.
*Stephanorhinus* sp. (cf. *S. hemitoechus*)	*Stephanorhinus* cf. *S. hemitoechus*
*Hippopotamus sp.?Megaloceros* sp.	*Hippopotamus* cf. *H. amphibius*
*Cervus elaphus*	*Megaloceros* cf. *M. giganteus*
*Cervus* vel *Dama*	*Cervus elaphus*
*Dama dama*	*Dama dama dama*
*Capreolus capreolus*	*Dama dama* cf. *D. d. tiberina*
*Bos primigenius*	*Capreolus capreolus*
Caprini ind.	*Bos primigenius*

### Elephantidae

#### Palaeoloxodon antiquus

The presence of the straight-tusked elephant is indicated by several upper and lower molars, and numerous fragments of ribs and vertebrae, and short and long limb-bones, referable to young and adult individuals.

#### Mammuthus ex gr. chosaricus-primigenius

Bones and dental remains of *Mammuthus* also occur, characterized by the rather archaic structure of the molars, testified by the enamel thickness (unusual for *M. primigenius*) and by the rather broad lamellar frequency, typical of the *trogontherii-chosaricus-primigenius* transitional forms^22–24^.

### Ursidae

#### Ursus spelaeus

This species is represented by several fossil remains. The shape and size of the limb-bone fragments and especially of the upper canine are suggestive of this species rather than *U. deningeri* or *U. arctos*^25,26^.

### Canidae

#### Canis lupus

A mandibular fragment of modest size with a second lower molar, probably belonging to a female^27^, testifies to the presence of this species at Campoverde.

### Equidae

#### Equus ferus

Numerous teeth and limb-bone remains can be attributed, from morphology and large dimensions, to *E. ferus*. Upper molars and premolars display the plis caballin and symmetric and lengthened protocone; the lower cheek teeth show the lingual flexid of caballine-type and, as for the upper teeth, the plis caballin^28^ (with references).

#### Equus hydruntinus

Some upper teeth and a fragmentary metacarpus belong to this small-sized equid. Upper molars display interstylar faces with a flattened trend, simple pilasters and short protocone with the typical shape of “horse hoof”^28^.

### Rhinoceratidae

#### Stephanorhinus sp

Many fragments of upper and lower teeth and some fragmented post-cranial bone remains of Rhinoceratidae can be referred generically to genus *Stephanorhinus*. Among these, one was probably worked to realize a bone tool.

A fragmentary upper premolar is brachydont and it shows a rather rough enamel, a marked cingulum and occlusial surface with contours similar to those of *S*. *hemitoechus*^29^, and it can be prudently attributed to *Stephanorhinus* cf. *S*. *hemitoechus*.

### Hippopotamidae

#### Hippopotamus cf. H. amphibius

A fragmented humerus can be attributed to *Hippopotamus* cf. *H. amphibius* from its small size, compatible with the dimensions of the species^30^.

### Cervidae

#### Megaloceros cf. M. giganteus

A few specimens, including two complete astragals, show the typical morphology of the Cervidae, and belong to a large cervid, probably the giant deer.

#### Cervus elaphus

The presence of red deer is indicated by several fragmented cranial and post-cranial bones, including some upper and lower teeth. A third lower molar belonging to an elderly individual display the very large third lobe almost completely combined with the other two. These morphological features and the dimensions (L. 28.90, B. 12.70 mm) are close to the modern forms of *C. elaphus*, which occurred in the peninsula at the onset of Late Pleistocene^31–33^.

#### Dama dama ssp

**Figure 4:**
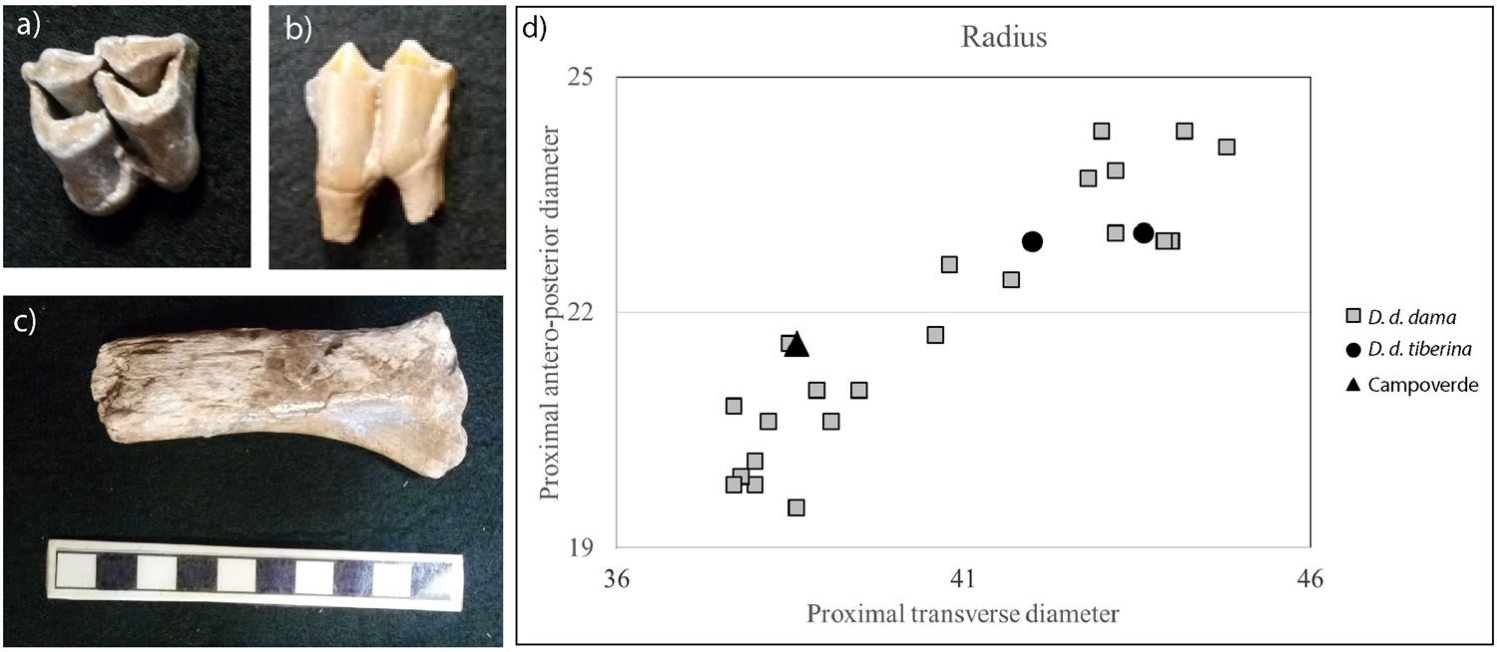
(**a**) Upper and (**b**) lower molar, and (**c**) proximal radius of *Dama dama dama* (photos by authors). (**d**) Scatter diagram of major measurements (mm) of the fallow deer subspecies proximal radius.

Several cranial and post-cranial remains belong to the fallow deer. An upper molar shows a weak anterior and posterior cingulum, but does not display the presence of any entostyle (Fig. 4a); a rather small lower molar (L. 17.80, B. 10.0 mm) displays an imperceptible anterior cingulum and, above all, the absence of the entostyle (Fig. 4b). These morphological features permit attribution of these teeth to the recent form of modern fallow deer, *D. dama dama*^32^. A particularly thin diaphysis of metatarsus and a proximal radius (Fig. 4c,d) of reduced size can also be attributed to this subspecies.

Some post-cranial remains have the typical morphology of fallow deer^34^, but the sizes are larger than the current *D. dama dama* and are unusual also for the Late Pleistocene fallow deer (e.g., metacarpus: MTD 21.5, MAPD 20.5 mm; tibia: DTD 36.0, DAPD 30.9 mm). Probably, these bones belong to the archaic form of modern fallow deer, *D. dama tiberina* (see^32^), and can be prudently referred to *D. dama* cf. *D. d. tiberina*.

#### Capreolus capreolus

A couple of brachydont lower check-teeth with very small sizes can be referred to *C. capreolus*.

### Bovidae

#### Bos primigenius

The aurochs is the most abundant taxon at Campoverde, as in the most of the Aurelian mammal assemblages^35^. The measurements of many specimens reveal a population of large size and some skeletal elements are larger than those of *B. primigenius* from Avetrana (Taranto, Apulia), from a fossiliferous deposit referred to MIS 5^36^. For example, a metacarpus 169 mm long and belonging to a male (DB/L and MB/L indices = 35.53 and 20.82, respectively) was estimatedto have a withers height^37^ of 170.3 cm, among the largest recorded in Italy.

## The Lithic Assemblage

**Figure 5:**
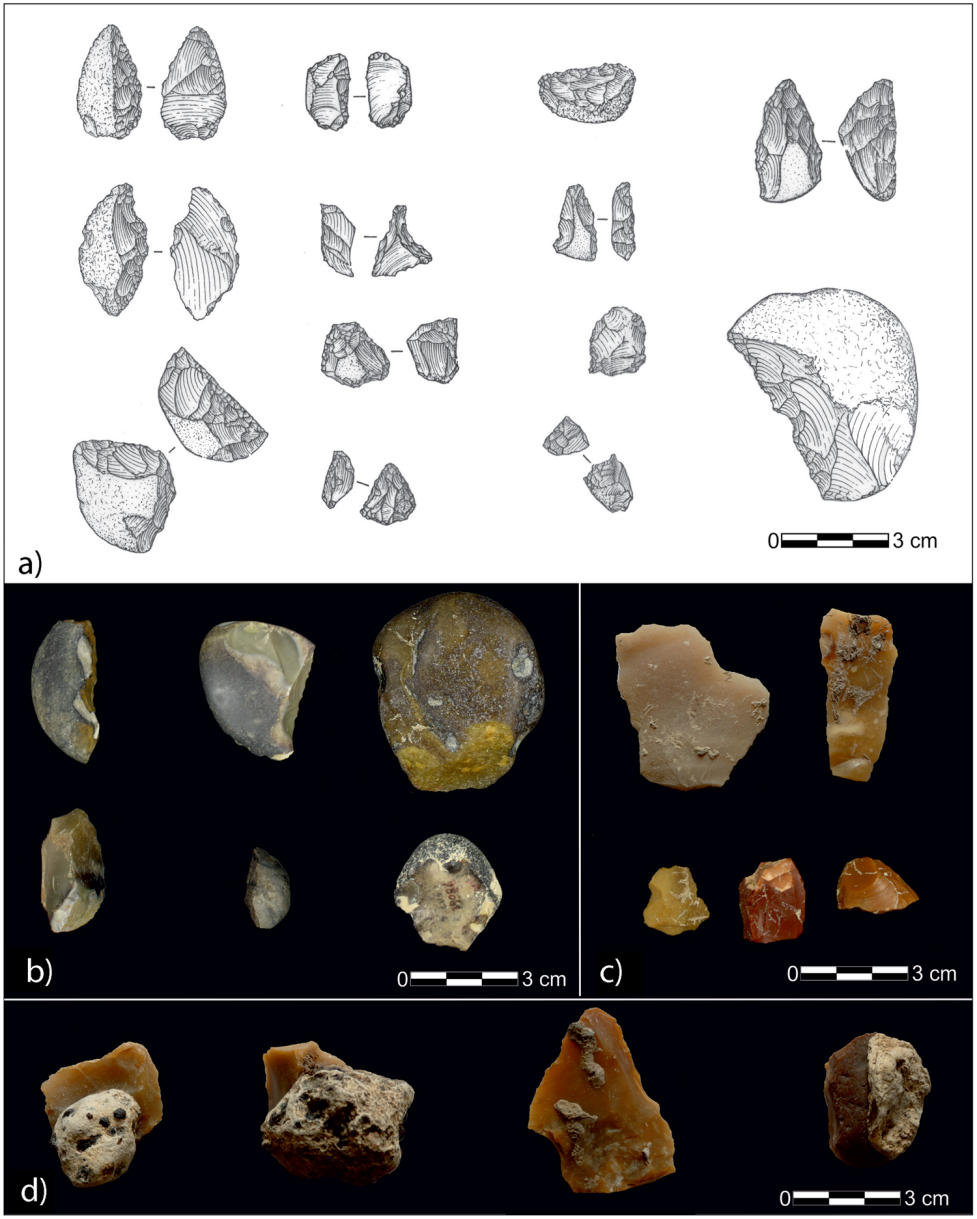
(**a**) Lithic industry from the first technocomplex (re-drawn here from original drawings by^2^). (**b**) Artifacts displaying the brownish polished patina (authors image). (**c**) Ichnotraces by “vermetidae”, identified on few artifacts (authors image). (**d**) Artifacts displaying carbonate concretions (authors image).

The lithic industries studied in the late 1990s and early 2000s belong to a technocomplex with a high percentage of corticated blanks obtained from small pebbles (<5 cm), a strong predominance of corticated and smooth striking platform, an absence of Levallois technique, and the presence of bipolar and centripetal technology of cores (Fig. 5a ^2–4^). Unretouched blanks are rare whilst carinated blanks are abundant. Retouched blanks are extremely small and are mainly composed of denticulates, often microlithic (42,8%), side scrapers (21,1%), borers (8,8%), other tools (7,5%), end scrapers (6,8%) and choppers (6,1%) (Fig. 5a). These characteristics compare well with the lithic assemblage from Casal De’ Pazzi^3,38^, therefore suggesting an MIS 7 age^39^.

A second collection of 317 implements from Campoverde has been recently analysed for this study. The main features are the preponderance of unretouched blanks (=179), retouched blanks (=70), cores (=26) and debris (=42). A large number of side scrapers (76%), notches and denticulates (13%) and rare borers (2%) were identified. The Levallois technique is rarely observed (3%). Cores are perfectly distributed among centripetal (23%), prepared platform (23%) and shapeless or exhausted (23%), while pseudoprismatic (11,5%), globular (8%) flaked pebbles (8%) and a single chopper core (3,5%) are identified.

The first assemblage displays diverse and interesting taphonomic evidence. A large number of artifacts exhibit a brownish polished patina (86%; Fig. 5b), a small percentage (14%) does not display no patina at all, while sharp and rounded edges are present in both categories. Moreover, ichnotraces (Fig. 5c) and encrustations (Fig. 5d) are present on a small number of implements. The ichnotraces are probably attributable to encrusting aquatic organisms, probably *vermitidae*, and suggest that the area experienced marine conditions after the lithics were discarded. The encrustations seem to support a wet habitat, since these are identified as carbonate concretions with nodules and/or crystals of manganese (or iron), and strongly suggest that these specimens come from the whitish layer of carbonate lagoon mud. Notably, the second assemblage does not show patinas or encrustation of any type.

Taphonomic analyses suggest that different depositional dynamics characterise the industries composing this collection, which certainly reached the site from diverse source areas, likely at some distance for those implements with rounded edges. Otherwise from specimens with the brownish polished patina, which point to a prolonged emplacement within a peat deposit, those without patina were most probably discarded in the area in a period of time when it was characterized by a different environmental/geological framework. Some of the brownish specimens display both ichnotraces and carbonate encrustations. Remarkably, while the first assemblage features diverse taphonomy, suggesting extremely disparate and problematic depositional dynamics, the second assemblage presents an extended homogeneity indicating an almost contemporary deposition of the artifacts.

## Discussion

**Figure 6:**
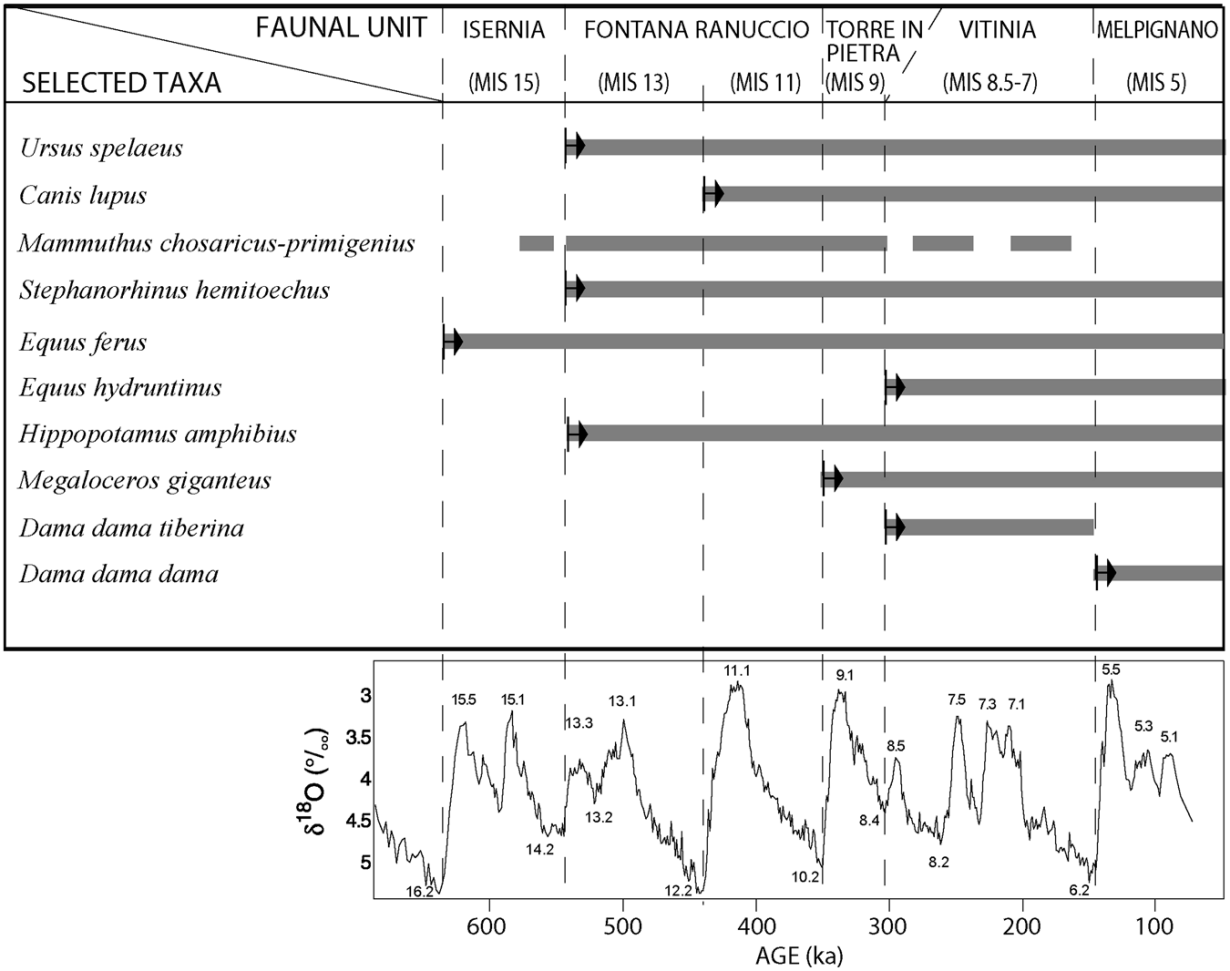
Biochronological scheme showing the range and the FO (black arrows) of the principal taxa constituting the faunal assemblage of Campoverde. Comparison with the Oxygen Isotope timescale^12^ is also shown.

[1] pointed out that the exact stratigraphic position within the sedimentary succession of the majority of the finds is unknown, and that many fossil bones show evidence of transportation, suggesting that several of these may be reworked. Nevertheless, these remains were considered as a single assemblage, referred it to middle-late Middle Pleistocene^1^. In fact, most of the taxa found at Campoverde (such as *Palaeoloxodon antiquus*, *Ursus spelaeus*, *Canis lupus*, *Equus ferus*, *Stephanorhinus hemitoechus*, *Hippopotamus amphibius, Bos primigenius*, and *Capreolus capreolus*) have their First Occurrence (FO) in Italy in the Galerian, and are well represented in the Aurelian mammal assemblages^35^, spanning a wide temporal range (MIS 15/13 through MIS 7, i.e.: 600–200 ka, see Fig. 6).

Similarly, *Megaloceros giganteus* is reported in Italy from MIS 9, in the Torre in Pietra Faunal Unit (FU), becoming quite frequent in the Late Pleistocene and disappearing at the end of the Last Glacial^40,41^. *Equus hydruntinus*, which was firstly recorded in the Vitinia FU, MIS 8.5–7, became more common during the Late Pleistocene, and became extinct during the middle Holocene^35,42^. Also *Cervus elaphus*, which occurs for the first time in the Slivia FU, MIS 19, and is still living in Italy, does not offer biochronological information, because such evidence requires identification at the subspecies level only, and can be made mostly by comparison of the antlers^31,33^, which are not reported among the fossil remains of Campoverde.

In contrast, discriminating elements are represented by *Mammuthus* and *Dama dama*. Indeed, if *M. primigenius* occurs in Italy in the Late Pleistocene, the transitional forms *M. trogontherii-chosaricus-primigenius* are recorded only from the late Galerian to middle-late Aurelian, in the Middle Pleistocene^23,24,35,41^ (Fig. 6). Among the modern forms of fallow deer, *D. dama tiberina* occurs only in the Vitinia FU, MIS 8.5–7, while *D. dama dama* occurs at the beginning of Late Pleistocene, and was particularly abundant in central-southern Italy during the last interglacial, and then progressively decreased in abundance before extinction in MIS 2^32,41^.

The presence at Campoverde of *D. dama dama*, which occurred only in the Late Pleistocene, and of *Mammuthus* ex gr. *chosaricus-primigenius*, recorded only in the Middle Pleistocene, clearly indicates two distinct mammal assemblages. The younger one is referred to the Late Pleistocene, and should be considered to reflect the age of deposition of the aggradational succession forming the Campoverde complex. This transgressive deposit forms a marine terrace, at an elevation ranging 31–40 m, that we correlate with MIS 5e. An older faunal assemblage is referred to the late Middle Pleistocene; the occurrence of *E. hydruntinus* along with the possible occurrence of *D. dama tiberina*, constraints the time interval to the Vitinia FU, MIS 8.5–7^35^ (Fig. 6). We interpret it as evidencing the occurrence of reworked material within the basal coarse deposit, incorporating misplaced fossils eroded from the older deposits forming the higher terrace of MIS 7 age, whose remnants are recognized in the northwestern sector of the investigated area, at an elevation of 51–58 m a.s.l. (Fig. 2a).

We remark that amino acid geochronology for the sand deposits at an elevation of ~12 m a.s.l. in the Agro Pontino (Minturno complex) provided two groups of Alle/Ile ratios (site 15 in^43^). One considered typical of MIS 5e (0.39, within the E aminozone), and another, dominant group indicative of a later stage 5 age (0.29, within the C/D aminozone). These data suggest the occurrence of reworked, older shells from the MIS 5e terrace conglobated within the deposit of a younger transgressive cycle, according to the correlation proposed in this paper with MIS 5a for the Minturno complex.

Consistently, the techno-typological study supports interpretation of the collection recovered at Campoverde as composed of two different technocomplexes, with the first assemblage highlighting a predominance of denticulates whilst the second is mainly composed of side scrapers. Taphonomic and techno-typological characteristics suggest a different chronological attribution of the assemblages which, however, can be generally assigned to the MIS 7–5a timeframe.

## Conclusion and Final Remarks

The correlation of the 36 m terrace with MIS 5e in the investigated area south of Anzio is consistent with the wider regional geomorphological setting, where this terrace is continuously recognized in the coastal area between Civitavecchia and Anzio, and is geochronologically constrained at 129 ka (see Fig. 1b). Based on this straightforward correlation, the two lower terraces at ~26 and ~15 m in the Agro Pontino must be correlated with younger interstadials, namely MIS 5c and 5a, respectively, as already proposed for the equivalent terraces reconstructed in the central Latium coast by^5^. As a consequence, previous correlation of the lowest terrace (Minturno complex) and of the related sea-level indicators between 12 and 10 m a.s.l. with MIS 5e^15^ appears questionable, and should be revised.

Notably, this area was previously considered tectonically stable and the sea-level elevation of ca. 9 m testified by tidal notches in the Circeo promontory^44,45^ has been considered a benchmark for estimation of the maximum amplitude of the sea-level oscillation during MIS 5e in the Mediterranean Sea (e.g.^46^). We believe that the new data presented in this paper require reconsideration of the sea-level history during the Last Interglacial Stage for the central Mediterranean Sea so far conceived, as well as, provide new insights on the tectonic history of the Tyrrhenian Sea Margin of central Italy, evidencing a uniform uplift affecting the whole coastal reach of this region since 125 ka. However, no safe geochronologic constraint to this sea-level exists in the literature, and future work needs to be addressed aimed at providing direct dating of the associated deposits, as well as, of those associated with the 26 m and with the 36 m terraces in this area.

## Data Availability

All data generated or analysed during this study are included in this published article.
